# Early Diagnosis of Acute Appendicitis in the Second Trimester of Pregnancy Based on Non-typical Clinical Findings: Report of a Rare Case and a Mini-Review of the Literature

**DOI:** 10.7759/cureus.61463

**Published:** 2024-05-31

**Authors:** Anna Thanasa, Efthymia Thanasa, Ioannis-Rafail Antoniou, Alexandros Leroutsos, Ektoras-Evangelos Gerokostas, Gerasimos Kontogeorgis, Vasileios Papadoulis, Athanasios Ntavanos, Ioannis Paraoulakis, Ioannis Thanasas

**Affiliations:** 1 Department of Health Sciences, Medical School, Aristotle University of Thessaloniki, Thessaloniki, GRC; 2 Department of Obstetrics and Gynecology, General Hospital of Trikala, Trikala, GRC; 3 Department of Anesthesiology, General Hospital of Trikala, Trikala, GRC; 4 Departament of Anesthesiology, Genaral Hospital of Trikala, Trikala, GRC; 5 Department of Obstetrics and Gynecology, General Hospial of Trikala, Trikala, GRC

**Keywords:** acute appendicitis, pregnancy, clinical presentation, ultrasound, magnetic resonance imaging, appendectomy, case report

## Abstract

Acute appendicitis is the most common non-obstetric reason for exploratory laparotomy during pregnancy. This case report involves a primigravida patient who presented to the emergency department of the General Hospital of Trikala at 15 weeks of gestation due to diffuse abdominal pain, primarily in the epigastric region. She also reported watery bowel movements. The ongoing atypical clinical symptoms, along with elevated inflammatory markers, strongly indicated a diagnosis of acute appendicitis. An immediate exploratory laparotomy was performed, during which acute localized inflammation of the appendix was found, leading to an appendectomy. Histological examination confirmed the diagnosis of acute appendicitis. The patient reported pain relief immediately after the surgery. On the fourth postoperative day, she was discharged without any signs of a threatened second-trimester miscarriage. At 39 gestational weeks, she delivered by elective cesarean section due to breech presentation. This paper discusses the case and highlights the significant challenges in the early diagnosis and management of acute appendicitis during pregnancy, emphasizing the importance of preventing potentially life-threatening complications for both the mother and the fetus.

## Introduction

Acute abdomen in pregnancy is rare but clinically significant. It is characterized by severe abdominal pain lasting less than 24 hours and may necessitate emergency surgery. The physiological, anatomical, morphological, functional, hemodynamic, and biochemical changes that occur in a pregnant woman's body make the early diagnosis, management, and accurate prognosis of acute surgical conditions particularly challenging [1]. The incidence of acute abdomen in pregnancy is approximately one in every 500 to 635 pregnancies [2]. Acute appendicitis is the most common non-obstetric cause of acute surgical abdomen in pregnant women, accounting for 65.6% of non-traumatic surgical emergencies in this group [3]. It affects about one in 700 to 4,000 pregnancies and is most frequently seen in the second trimester [4]. The prognosis for acute appendicitis worsens with advancing gestational age and a delay in diagnosis. In complicated cases of appendicitis, such as appendiceal abscess, gangrenous appendicitis, or appendiceal perforation, delayed diagnosis and treatment greatly increase the risk of serious complications for both the mother and fetus [5]. The risk of preterm delivery and fetal death in such complicated cases is 11% and 6%, respectively, compared to 6% and 2% in pregnant women with uncomplicated appendicitis [6].

This paper describes a rare case of acute appendicitis with an atypical clinical presentation in the early second trimester of pregnancy. It also addresses the significant diagnostic challenges associated with the early diagnosis and management of these patients. Emphasis is placed on the importance of early diagnosis, which is crucial for reducing maternal and perinatal morbidity and mortality.

## Case presentation

**Figure 1:**
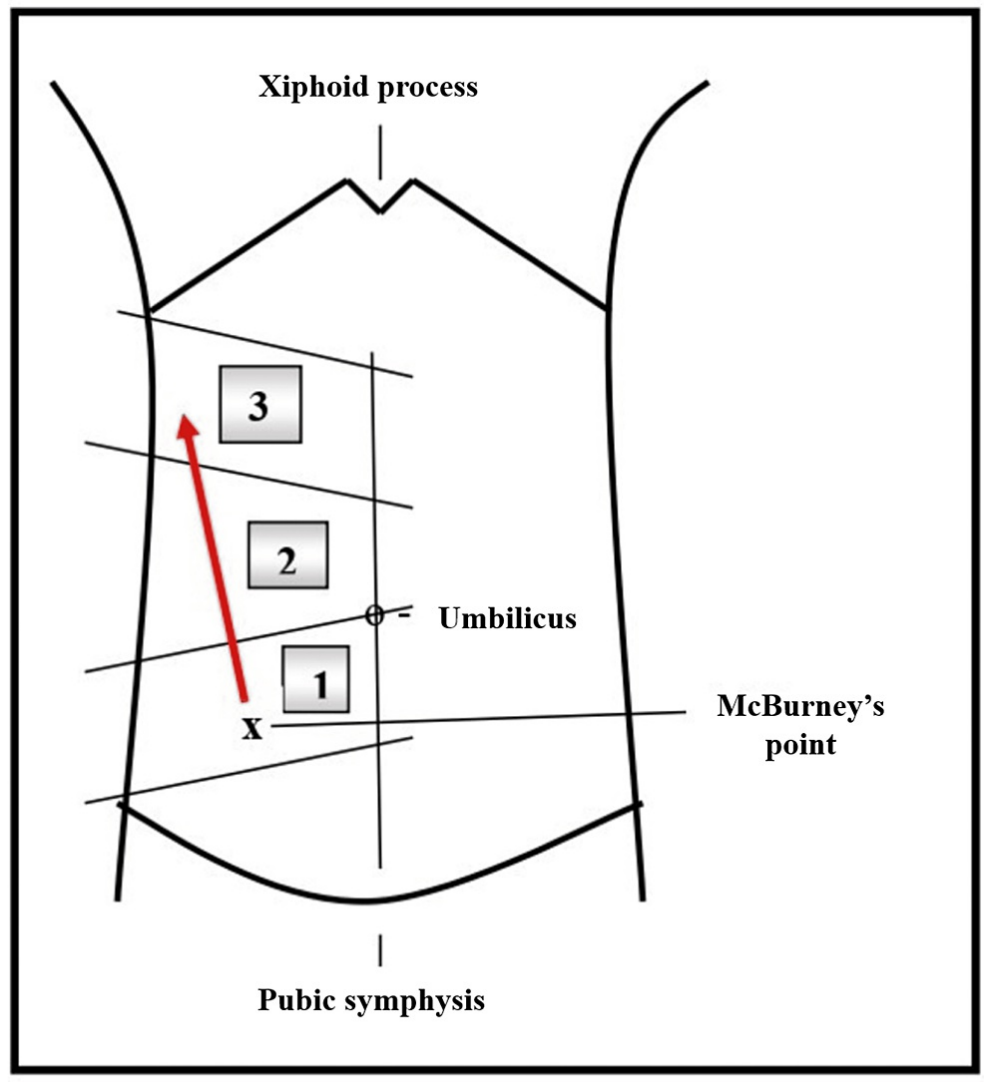
The image illustrates the displacement of McBurney's point on the right lateral abdominal wall (indicated by the red arrow) relative to gestational age in patients diagnosed with acute appendicitis. In the non-pregnant woman, McBurney's point (x) typically corresponds to the outer third of an imaginary line drawn from the umbilicus to the right superior anterior iliac spine; in pregnant individuals, the area of tenderness for acute appendicitis shifts as pregnancy progresses: one for the first trimester, two for the second trimester, and three for the third trimester. This image has been created by the authors.

This case report involves a 21-year-old primigravida woman who presented to the emergency department of the General Hospital of Trikala at 15 gestational weeks. She reported diffuse abdominal pain for approximately five hours, primarily in the epigastric region. She also mentioned having two diarrheal bowel movements following the onset of the pain. The pain was not associated with anorexia, nausea, or vomiting. Her medical history was unremarkable, and her obstetric follow-up indicated a normal pregnancy up to that point. During the physical examination, her body temperature was normal (36.8 degrees Celsius), and her blood pressure and pulse rate were within normal limits (110/70 mmHg and 91 beats per minute, respectively). Abdominal palpation revealed tenderness throughout the abdomen, particularly in the epigastric region, without signs of peritoneal irritation or rebound tenderness. Additionally, the characteristic displacement of McBurney's point on the right lateral abdominal wall at the level of the umbilicus, commonly associated with acute appendicitis, was not observed (Figure 1).

An obstetric ultrasound, performed after the admission of the patient, showed no signs of threatened miscarriage, such as placental abruption or subchorionic hematoma. Ultrasound examination of the upper and lower abdominal regions also revealed no abnormalities. The liver was normal in size with normal echogenicity and no evidence of intrahepatic or extrahepatic bile duct dilatation was observed. The pancreas appeared normal, and the imaging of the kidneys, ureters, and bladder was unremarkable. However, ultrasound imaging of the appendix was not possible due to bowel air congestion. Emergency laboratory tests indicated elevated inflammatory markers (Table 1).

**Table 1:** Laboratory control of the patient during her hospitalization at the clinic Ht: hematocrit; HB: hemoglobin; PLT: platelets; WBC: white blood cells; NEUT: neutral; APTT: activated partial thromboplastin time; INR: international normalized ratio; CRP: C reactive protein; Glu: glucose; CR: creatinine; Na+: sodium; K+: potassium; TBIL: total bilirubin; DBIL: direct bilirubin; ΙDBIL: indirect bilirubin

Laboratory tests	Day of admission to the clinic	12 hours of hospitalization	Third postoperative day after appendectomy	Normal laboratory values
Ht	35.1%	34.3%	33.1%	37.7% – 49.7%
Hb	11.6 gr/dl	11.1 gr/dl	10.7 gr/dl	11.8 – 17.8 gr/dl
PLT	225x10^3^/ml	207x10^3^/ml	215x10^3^/ml	150 – 350 x10^3^/ml
WBC	15.9x10^3^/ml	16.1x10^3^/ml	9.3x10^3^/ml	4 – 10.8 x10^3^/ml
NEUT	84%	85%	71%	40 – 75%
APTT	29.3 sec	30.5 sec	29.7 sec	24.0 – 35.0 sec
INR	0.97	1.01	0.95	0.8 – 1.2
CRP	11.7 mg/dl	16.9 mg/dL	1.1 mg/dL	0.5 mg/dl
Glu	97 mg/dl			75 – 115 mg/dl
Cr	0.59 mg/dl			0.40 – 1.10 mg/dl
Na^+^	139mmol/L			136 – 145 mmol/L
K^+^	4.1mmol/L			3.5 – 5.1mmol/L
TBIL	0.41 mg/dl	0.42 mg/dl		0 – 1.2 mg/dl
DBIL	0.30 mg/dl	0.25 mg/dl		0 – 0.5 mg/dl
ΙDBIL	0.11 mg/dl	0.17 mg/dl		0 – 0.7 mg/dl
Amylase	78 U/mL			0 – 110 U/mL

Liver function tests and serum and urine amylase levels were within normal limits, and general urinalysis showed no abnormalities. Stool culture results were negative. A clinical examination by a surgical team could not rule out acute appendicitis. Due to the lack of emergency magnetic resonance imaging (MRI) at our hospital and the decision to avoid computed tomography (CT) to prevent fetal exposure to ionizing radiation, the patient was closely monitored and treated with antibiotics, given the high suspicion of acute appendicitis.

The persistence of atypical clinical findings, along with elevated inflammatory markers on repeat laboratory tests after 12 hours (Table 1), further reinforced the suspicion of acute appendicitis. After discussing the risks of delaying treatment with the patient and her family, an immediate exploratory laparotomy with a right lateral incision was decided. A laparoscopic approach was not feasible due to the surgical team's lack of experience. Intraoperatively, an inflamed appendix was identified, and an appendectomy was performed. Histological examination confirmed the diagnosis of acute appendicitis. The patient reported immediate pain relief postoperatively. With no signs of second-trimester miscarriage, she was discharged on the fourth postoperative day with instructions for follow-up at the outpatient clinic. Upon reaching 39 gestational weeks, the patient delivered by scheduled cesarean section due to a breech presentation.

## Discussion

Diagnosing acute appendicitis during pregnancy can be challenging. The common symptoms (abdominal pain, fever, nausea, vomiting, and anorexia) are non-specific and often resemble those of a normal pregnancy or the symptoms of other pathological conditions, such as urinary tract infection, gastroenteritis, or gynecological emergencies like adnexal torsion [7,8]. During a clinical examination, palpation usually reveals a shift in the point of tenderness, known as McBurney's point, to the right lateral abdominal wall. This shift occurs due to the anatomical changes during pregnancy that progressively move the cecum and appendix upward, rightward, and backward, depending on the trimester (Figure 1). Consequently, the location of pain varies with each trimester. In the third trimester, pain may be felt in the right upper quadrant of the abdomen. In the second trimester, pain is generally located in the right lateral abdominal region at the level of the umbilicus. In the first trimester, the pain is typically in the right lower quadrant of the abdomen, similar to that in non-pregnant patients [9]. Our patient, who was in the 15^th^ week of pregnancy, reported diffuse abdominal pain primarily located in the epigastric region, along with diarrheal bowel movements. The pain was not situated in the right abdomen or at the level of the umbilicus, which would typically be expected in the second trimester. Additionally, the pain was not accompanied by the typical clinical symptoms of acute appendicitis, such as fever, anorexia, nausea, and vomiting.

Additionally, the typical biochemical and laboratory markers used to diagnose acute appendicitis may be less reliable in pregnant patients. Elevated white blood cell counts, increased erythrocyte sedimentation rates, and C-reactive protein levels above the normal range should be cautiously interpreted in pregnant women, as these can occur in a normally progressing pregnancy. "Normal" leukocytosis during pregnancy can reach up to 16,000/ml, complicating early diagnosis since white blood cell counts in acute appendicitis cases do not always exceed this threshold [10]. However, an increase in neutrophil percentage to ≥85.30% and a rise in C-reactive protein to ≥34.26 mg/L in the third trimester strongly suggest the presence of complicated appendicitis [11]. Numerous studies have investigated the role of preoperative inflammatory markers in supporting the clinical diagnosis of acute appendicitis during pregnancy. In 2022, Peksöz et al. suggested that white blood cell count, neutrophil percentage, neutrophil-to-lymphocyte ratio (NLR), platelet-to-lymphocyte ratio, and total, direct, and indirect bilirubin levels could aid in diagnosing acute appendicitis in pregnant women and may even predict complicated forms of the disease in some cases [12]. In 2023, Feng et al. conducted a meta-analysis and found that the NLR is a valuable and effective diagnostic tool for acute appendicitis during pregnancy [13]. More recently, in 2024, Adir et al. demonstrated that biomarkers such as the NLR and the monocyte-to-lymphocyte ratio (MLR) can be useful for diagnosing acute appendicitis, particularly in pregnant women where the use of CT is contraindicated [14].

The contraindication to using CT during pregnancy due to the risk of fetal exposure to ionizing radiation complicates the early diagnosis of acute appendicitis, despite CT being the most useful and effective diagnostic imaging modality [15,16]. Low-dose CT might be considered when the diagnosis of acute appendicitis cannot be established by ultrasound or MRI [17]. Ultrasound is the first-line imaging modality for diagnosing acute appendicitis during pregnancy [18]. Unlike CT, ultrasonography does not use ionizing radiation, making it a safe and readily available option, though it has moderate sensitivity for detecting an inflamed appendix in pregnant patients [19]. An MRI is an excellent alternative for pregnant women with suspected acute appendicitis, as it does not expose the fetus or mother to ionizing radiation. With high sensitivity and specificity (91.8% and 97.9%, respectively), MRI is expected to play a significant role in the differential diagnosis of acute abdominal pain in pregnancy, particularly when ultrasound results are inconclusive, potentially replacing the need for CT, which poses risks to the fetus [20, 21]. Recently, in 2023, Le et al. demonstrated that MRI findings, such as an increase in appendiceal diameter and appendiceal wall thickness, significantly aid in the early diagnosis of acute appendicitis in pregnant women [22]. In our case, a CT scan was not used, and an MRI was not available for emergency cases at our hospital. The ultrasound results were inconclusive. Therefore, the diagnosis of acute appendicitis and the decision to perform an immediate appendectomy were based on the atypical clinical findings and the persistence of elevated inflammatory markers.

Conservative treatment is not considered appropriate for treating appendicitis during pregnancy. Unlike surgery, conservative treatment is associated with a significantly higher risk of septic shock and peritonitis [23]. A recent study by Cheng et al., published in 2023, showed that while the trend for non-surgical management of pregnant women with uncomplicated appendicitis is increasing, the clinical outcomes are worse compared to surgical management [24]. Therefore, immediate appendectomy is the preferred treatment option for suspected acute appendicitis in pregnancy [25]. In 2007, Yilmaz et al. demonstrated that the time interval from the onset of symptoms to surgery (whether laparoscopy or laparotomy) should not exceed 20 hours to ensure optimal maternal and fetal well-being [26]. The laparoscopic technique for acute appendicitis in pregnancy offers several key advantages over open laparotomy, including the use of milder anesthetics, which reduces the risk of fetal distress, shorter hospitalization time, reduced postoperative pain, a better intraoperative field of view, faster recovery of gastrointestinal function, and quicker patient mobilization [27]. According to the most recent recommendations from the Society of American Gastrointestinal and Endoscopic Surgeons (2024), the laparoscopic approach is also preferred in the late stages of pregnancy [28]. In our patient's case, an exploratory laparotomy was promptly decided. Open appendectomy was chosen due to the surgical team's lack of experience with laparoscopic appendectomy.

## Conclusions

Acute appendicitis stands as the leading cause of non-obstetric acute abdomen during pregnancy. Given the alterations in the disease's clinical progression and pregnant women's physical examination, a meticulous multidisciplinary approach is imperative. When encountering atypical clinical presentations that cannot be attributed to other medical conditions, suspicion for acute appendicitis should be heightened. Timely diagnosis and management are paramount, as delays can result in severe complications affecting both mother and fetus. The decision for immediate surgical intervention should be considered, even in cases where diagnostic findings are not typical. Further research endeavors aimed at enhancing the accuracy of diagnosing acute appendicitis during pregnancy are warranted.
